# Predicting Extrathyroidal Extension in Papillary Thyroid Carcinoma Using a Clinical-Radiomics Nomogram Based on B-Mode and Contrast-Enhanced Ultrasound

**DOI:** 10.3390/diagnostics13101734

**Published:** 2023-05-13

**Authors:** Liqing Jiang, Shiyan Guo, Yongfeng Zhao, Zhe Cheng, Xinyu Zhong, Ping Zhou

**Affiliations:** 1 Department of Ultrasound, The Third Xiangya Hospital, Central South University, Changsha 410013, China; 2 Department of Oncology, NHC Key Laboratory of Cancer Proteomics, Laboratory of Structural Biology, National Clinical Research Center for Geriatric Disorders, Xiangya Hospital, Central South University, Changsha 410008, China

**Keywords:** papillary thyroid carcinoma, extrathyroidal extension, radiomics, b-mode ultrasound, contrast-enhanced ultrasound, nomogram

## Abstract

Papillary thyroid carcinoma (PTC) is the most common pathological type of thyroid cancer. PTC patients with extrathyroidal extension (ETE) are associated with poor prognoses. The preoperative accurate prediction of ETE is crucial for helping the surgeon decide on the surgical plan. This study aimed to establish a novel clinical-radiomics nomogram based on B-mode ultrasound (BMUS) and contrast-enhanced ultrasound (CEUS) for the prediction of ETE in PTC. A total of 216 patients with PTC between January 2018 and June 2020 were collected and divided into the training set (*n* = 152) and the validation set (*n* = 64). The least absolute shrinkage and selection operator (LASSO) algorithm was applied for radiomics feature selection. Univariate analysis was performed to find clinical risk factors for predicting ETE. The BMUS Radscore, CEUS Radscore, clinical model, and clinical-radiomics model were established using multivariate backward stepwise logistic regression (LR) based on BMUS radiomics features, CEUS radiomics features, clinical risk factors, and the combination of those features, respectively. The diagnostic efficacy of the models was assessed using receiver operating characteristic (ROC) curves and the DeLong test. The model with the best performance was then selected to develop a nomogram. The results show that the clinical-radiomics model, which is constructed by age, CEUS-reported ETE, BMUS Radscore, and CEUS Radscore, showed the best diagnostic efficiency in both the training set (AUC = 0.843) and validation set (AUC = 0.792). Moreover, a clinical-radiomics nomogram was established for easier clinical practices. The Hosmer–Lemeshow test and the calibration curves demonstrated satisfactory calibration. The decision curve analysis (DCA) showed that the clinical-radiomics nomogram had substantial clinical benefits. The clinical-radiomics nomogram constructed from the dual-modal ultrasound can be exploited as a promising tool for the pre-operative prediction of ETE in PTC.

## 1. Introduction

Papillary thyroid carcinoma (PTC) is the most common pathological type of thyroid cancer, and it has a good prognosis [1]; still, some PTCs develop aggressive biological behaviors such as lymph node metastasis (LNM) and extrathyroidal extension (ETE), which affect the recurrence rate and survival [2,3]. Li et al. found that the risk of recurrence in patients with ETE was 8.831 times higher than that in patients without ETE [4]. Ortiz et al. reported a 10-year survival rate of 99.3% in PTC patients without ETE, while the 10-year survival rate decreased to 63.2% in patients with ETE [5]. In addition, ETE is an important prognostic factor for PTC. Many prognostic risk stratification systems for differentiated thyroid cancer (DTC, PTC accounts for 85% of DTC [6]) use ETE as a prognostic indicator, such as the GAMES (gender, age, metastasis, extrathyroidal extension, size) system [7] and MACIS (metastases, age, completeness of resection, invasion, and size) system [8]. Currently, the histopathological result is still the “gold standard” for the diagnosis of ETE. According to the eighth edition of the AJCC-TNM staging system, ETE for thyroid carcinomas was divided into minimal and gross ETE. Minimal ETE is defined as the tumor invasion of the perithyroid soft tissue, while gross ETE refers to invasion into strap muscles and major neck structures (subcutaneous soft tissue, larynx, trachea, esophagus, recurrent laryngeal nerve, the prevertebral fascia, or it encases the carotid artery or mediastinal vessels) [9,10,11]. Surgery is the most effective means of routinely treating PTC. Many thyroid cancer scholars recommend that the appropriate decision for PTC patients at low risk is to perform an ipsilateral lobectomy, which will reduce postoperative complications such as recurrent laryngeal nerve injuries and hypoparathyroidism [12,13]. However, according to the National Comprehensive Cancer Network (NCCN) guidelines (Version 2.2022), ETE is considered to be a surgical indication for total thyroidectomy [14]. Therefore, the preoperative evaluation of ETE is crucial for helping the surgeon decide on the surgical plan.

The imaging methods used to evaluate ETE include magnetic resonance imaging (MRI), computed tomography (CT), and ultrasound (US). Compared with MRI and CT, US examination has become the preferred imaging method for preoperative thyroid tumor screening and prognosis assessments due to its advantages as follows: low cost, no ionizing radiation, real-time, and high resolution [15]. Contrast-enhanced ultrasound (CEUS) is a new technology that is widely used in clinical practice in recent years [16], which provides more reference information about microvascular perfusion for the evaluation of ETE [17]. Some studies have used some signs of conventional US and CEUS to evaluate ETE, such as the degree of contact between the thyroid tumor and capsule, and the disruption of capsule continuity [18,19,20,21,22]. However, these signs are subjective. In addition, ultrasound cannot reliably detect microscopic ETE, which can only be determined by postoperative pathology; thus, the sensitivity is relatively low [23].

Radiomics can excavate massive features that cannot be observed by the naked eye from medical images for the quantitative analysis of diseases [24,25,26,27]. It has been gradually applied in the research of various tumors, including tumor diagnosis, prognosis prediction, and gene analysis. Several studies have demonstrated the value of radiomics when applied in the differentiation of benign and malignant thyroid nodules [28,29,30] and the aggressiveness assessment of thyroid tumors [31,32,33,34,35]. For the preoperative prediction of ETE, Wang et al. constructed a US radiomics-based nomogram, which has a high predictive value [36]. However, compared with only using BMUS images, the addition of CEUS images can provide information on tumor vascularity and perfusion. Our previous studies have found that dual-modal US-based radiomics analyses can be used to distinguish benign from malignant thyroid nodules and also demonstrated that CEUS image-based radiomics analysis is promising in the study of PTC [28]. However, no previous studies have used CEUS-based radiomics to predict ETE in PTC patients. Therefore, this study attempted to explore the value of dual-modal radiomics features (radiomics features based on BMUS and CEUS images) and clinical risk factors in the pre-operative prediction of ETE. Then, we constructed a clinical-radiomics nomogram, and it is expected to be an effective clinical tool for the pre-operative prediction of ETE and assistance in the clinical treatment decision of PTC patients.

## 2. Materials and Methods

### 2.1. Patients

The protocol for this retrospective study was approved by the Institutional Review Board of the Third Xiangya Hospital of Central South University. The requirement for obtaining written informed consent from the patients was waived by the review board. Between January 2018 and June 2020, 216 patients in our hospital were retrospectively included. The study inclusion criteria were as follows: (1) BMUS and CEUS examinations were performed within 2 weeks before surgery; (2) postoperative histopathology confirmed the diagnosis of PTC and provided information on the ETE or non-ETE outcome; (3) a single unilateral lesion. The study exclusion criteria were as follows: (1) low-quality US images; (2) patients who received prior thyroidectomy; (3) incomplete clinical information and US image data. Using the postoperative histopathological results of the tumor as the gold standard, patients were classified into non-ETE and ETE groups.

### 2.2. BMUS and CEUS Examination

A GE LOGIQ E9 color Doppler ultrasonic instrument with a 9 L linear array probe was used to perform the US examination. The patient was placed in a supine position with full exposure of the neck. The radiologist performed a BMUS examination of the thyroid gland, identified the target nodule, and stored its maximum long-axis section image. After dissolving the freeze-dried SonoVue powder in 5 mL of normal saline, a suspension was formed after shaking and was prepared for use. After the maximum long-axis section of the target tumor was displayed; the probe was fixed; the machine was switched to CEUS mode; the mechanical index, focus, and gain were adjusted appropriately. A bolus injection of 2.4 mL of SonoVue contrast agent was administered through the cubital vein, followed immediately by the administration of 5 mL of normal saline. At the same time, the timing function and image storage were switched on, and the enhancement degree and capsule continuity of the thyroid nodules were observed. A frame of images corresponding to the peak time of CEUS was selected and stored for subsequent ROI delineation. Finally, BMUS images and CEUS images were exported in Dicom format.

### 2.3. Clinicoradiological Data Acquisition

Demographic characteristics including gender and age were obtained from the medical record system. Radiological features including site, size, internal echogenicity, aspect ratio, margins, calcifications, the enhancement degree, and US-reported ETE were determined and recorded by a radiologist with at least 15 years of experience while performing pre-operative ultrasound examinations. In BMUS images, tumors were considered to have BMUS-reported ETE (positive) when one or more of the following two features were observed: (1) >25% of the perimeter of the lesion was in contact with the thyroid capsule on BMUS; (2) discontinued capsule of the contact between the tumor and the thyroid gland on BMUS. In CEUS images, tumors were regarded as CEUS-reported ETE (positive) when there was a discontinued capsule enhancement of the contact between the tumor and the thyroid gland or an enhancement extending beyond the capsule.

### 2.4. Image Segmentation and Feature Extraction

**Figure 1:**
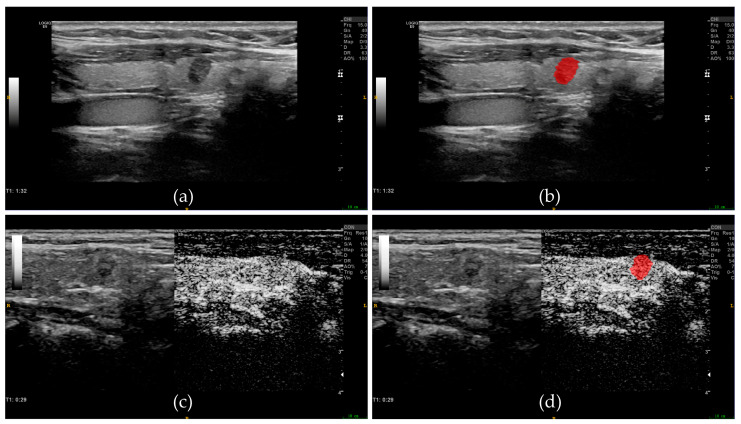
Examples of ROI segmentation on BMUS images and CEUS images. (**a**) BMUS image of the thyroid tumor. (**b**) Tumor ROI (red area) segmentation based on BMUS image. (**c**) CEUS image of the thyroid tumor. (**d**) Tumor ROI (red area) segmentation based on CEUS image. ROI, regions of interest; BMUS, B-mode ultrasound; CEUS, contrast-enhanced ultrasound.

The tumor region was segmented using ITK-SNAP software (version 3.8.0; http://www.itksnap.org/, accessed on 2 January 2021), and the radiologist manually delineated the border of the lesion and finally confirmed the region of interest (ROI), which included the entire area of the lesion as much as possible. BMUS images and CEUS images were used for ROI segmentation (Figure 1). The radiomics module of the 3D-Slicer software (version 4.11.20210226, https://www.slicer.org/, accessed on 1 October 2021) was used for radiomics feature extraction. All images were preprocessed before feature extraction, and this included resampling to a voxel size of 1 mm × 1 mm × 1 mm and image discretization with a fixed bin width of 25. In total, 837 radiomics features were extracted from each ROI, including 6 types of features: first-order features, gray-level dependence matrix (GLDM), gray-level co-occurrence matrix (GLCM), gray-level run length matrix (GLRLM), gray-level size zone matrix (GLSZM), and neighborhood gray-tone difference matrix (NGTDM). These features were extracted from the original image and the wavelet-transformed image, respectively. An interclass correlation coefficient (ICC) was used to assess inter-observer repeatability. Two radiologists (reader1 and reader2) performed ROI segmentation on the images of thirty randomly selected patients, and an ICC > 0.75 indicated good repeatability of radiomics feature extraction. One radiologist (reader1) completed all image segmentation.

### 2.5. Feature Selection and Model Construction

The total cases were divided into a training set and a validation set at a 7:3 ratio using a stratified random sampling method. Prior to feature selection, all radiomics features were normalized using the z-score normalization method. In the training set, only features with ICC > 0.75 were used for subsequent analysis. Then, the least absolute shrinkage and selection operator (LASSO) algorithm was used to further feature dimensionality reduction by ten-fold cross-validation. Finally, the optimal feature combination was selected by backward stepwise multivariate logistic regression analyses with the Akaike information criterion (AIC) to construct a model for calculating the radiomics score. The BMUS Radscore and CEUS Radscore were constructed based on BMUS images and CEUS images, respectively, by the above feature selection and model construction method.

Based on the training set, we first performed a univariate analysis of clinical factors (including demographic characteristics and radiological features) between the ETE and non-ETE groups. The statistically significant variables (*p* < 0.05) in univariate analysis were further analyzed using backward stepwise multivariate logistic regression to construct a clinical model.

The clinical risk factors with *p*-values < 0.05 in univariate analysis and two Radscores were included as candidate variables in backward stepwise multivariate logistic regression analysis to establish a clinical-radiomics model.

### 2.6. Model Comparison and Nomogram Development

We drew the receiver operating characteristic (ROC) curve and the area under the curve (AUC), and the DeLong test was used to evaluate and compare the predictive performance of the four models. A nomogram was then plotted to visualize the model with optimal performance for the ETE prediction of PTC. The Hosmer–Lemeshow test and calibration curves were used to evaluate the goodness of fit of the nomogram. The clinical utility of the nomogram was clarified by decision curve analysis (DCA).

### 2.7. Statistical Analysis

R software (version 4.1.3, https://www.r-project.org/, accessed on 1 November 2021) was used to load the corresponding functions or packages to complete the statistical analysis. Measurement data that fit the normal distribution were expressed as mean ± standard deviation, the measurement data that did not fit the normal distribution were expressed as medians (interquartile range), and the statistical significance of the difference between the two groups was determined by the *t*-test or Mann–Whitney U test. Enumeration data were expressed as n (%), and the chi-square test or Fisher’s exact test was used for comparisons between the two groups. *p* < 0.05 was considered statistically significant.

## 3. Results

### 3.1. Clinico-Pathological Information

**Figure 2:**
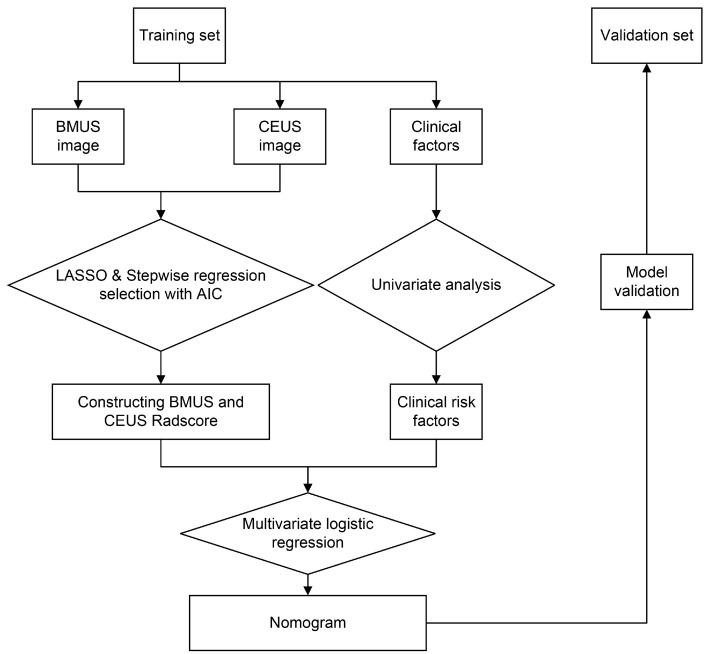
Flow chart of the study’s work. BMUS, B-mode ultrasound. CEUS, contrast-enhanced ultrasound. LASSO, least absolute shrinkage and selection operator. AIC, Akaike information criterion.

A study workflow diagram is shown in Figure 2. A total of 216 patients with PTC were included in this study. Pathological results showed that 70 patients had ETE and 146 patients had no ETE. These patients were divided into the training group (*n* = 152) and the validation group (*n* = 64) according to the random stratified sampling method. There were no statistically significant differences with respect to gender, age, site, tumor size, echogenicity, aspect ratio, margins, microcalcifications, enhancement degree, BMUS-reported ETE, and CEUS-reported ETE between the training and validation groups (all *p* > 0.05), as shown in Table 1, showing a balanced distribution of baseline patient characteristics between the training and validation sets. The positive rates of ETE were 33.6% and 29.7% in the training and validation groups, respectively, with no statistically significant difference (*p* = 0.579). The univariate analysis results of clinical parameters, BMUS Radscore, and CEUS Radscore in the training set and validation set are shown in Table 2.

### 3.2. Radiomics Scores

**Figure 3:**
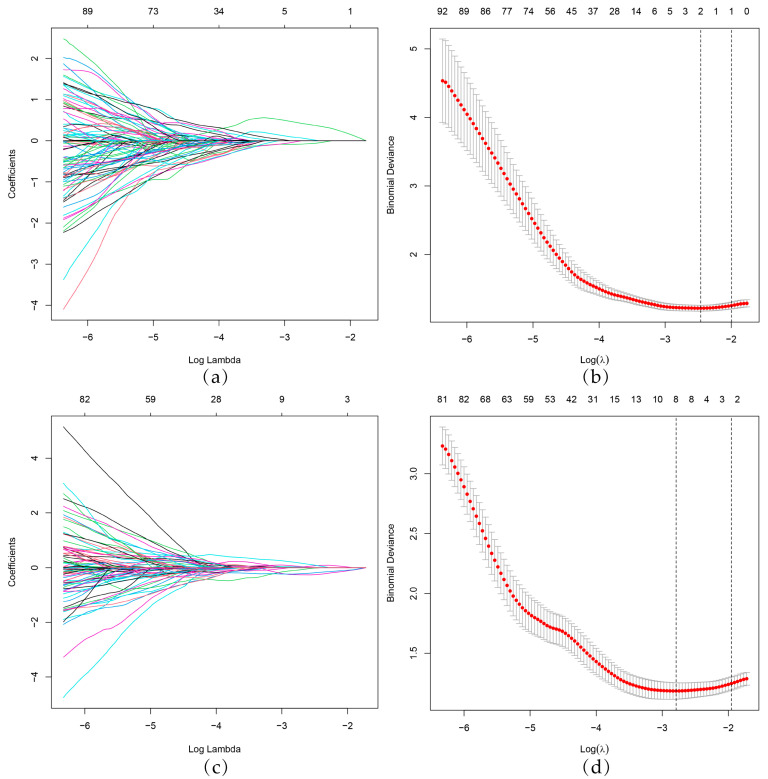
BMUS and CEUS radiomics feature selection by LASSO regression. (**a**,**c**) LASSO coefficient profiles of radiomics features. Each colored line represents the coefficient of an individual feature. (**b**,**d**) Selection of tuning parameters (lambda value) in the LASSO model using 10-fold cross-validation by the minimum criteria. Red dots indicate average deviance values for each model with a given λ, and vertical bars through the red dots show the upper and lower values of the deviances. The dotted vertical lines were drawn at the optimal λ value based on the minimum criteria and 1 standard error of the minimum criteria. BMUS, B-mode ultrasound. CEUS, contrast-enhanced ultrasound. LASSO, least absolute shrinkage and selection operator.

In total, 837 radiomics features were extracted from the ROI on the BMUS and CEUS images, respectively, and 774 BMUS and 783 CEUS radiomics features with ICC > 0.75 were retained. After the two-step dimensionality reduction via the Lasso algorithm (Figure 3) and backward stepwise logistic regression (Table 3), one BMUS feature was screened out and accordingly used to construct the BMUS radiomics model. The BMUS Radscore was calculated by summing the selected BMUS features weighted by their coefficients. Similarly, three CEUS features were selected via LASSO regression (Figure 3) and backward stepwise logistic regression (Table 3) for the CEUS radiomics model’s construction and CEUS Radscore calculation. The calculation formulas of the BMUS Radscore and the CEUS Radscore are shown below.

BMUS Radscore = −0.7010 + 0.9476 × original_ngtdm_Busyness

CEUS Radscore = −0.8609 + 0.6282 × wavelet.LHL_glszm_SmallAreaEmphasis − 0.6996 × wavelet.HLL_gldm_DependenceVariance + 0.5294 × wavelet.HLL_ngtdm_Complexity

**Figure 4:**
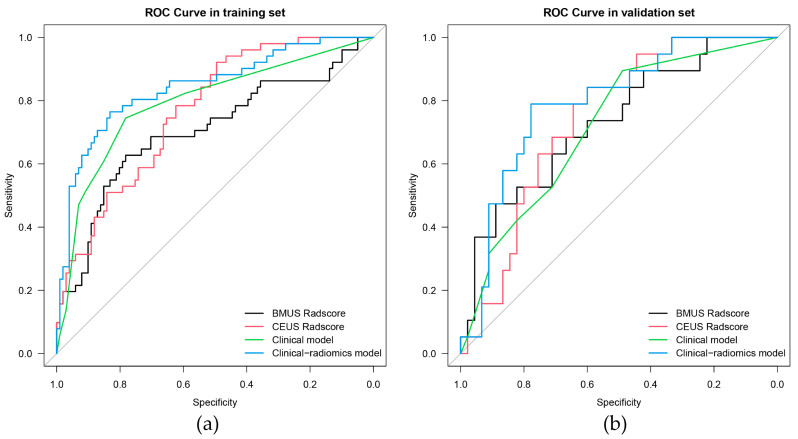
ROC curves of the BMUS Radscore (black lines), CEUS Radscore (red lines), clinical model (green lines), and clinical-radiomics model (blue lines) in the training (**a**) and validation (**b**) groups. ROC, Receiver operating characteristic. BMUS, B-mode ultrasound. CEUS, contrast-enhanced ultrasound.

The ROC curves are plotted in Figure 4. For predicting ETE, in the training set, the AUC of the BMUS Radscore was 0.704, with sensitivity and specificity values of 0.627 and 0.782, respectively; in contrast, in the validation set, the AUC was 0.731, with sensitivity and specificity values of 0.632 and 0.689, respectively. In the training set, the AUC of the CEUS Radscore was 0.768, with sensitivity and specificity values of 0.922 and 0.495, respectively; in contrast, in the validation set, the AUC was 0.739 with sensitivity and specificity values of 0.947 and 0.333, respectively (Table 4).

### 3.3. Clinical Model

In the training set, univariate analysis revealed statistically significant differences with respect to age, tumor size, BMUS-reported ETE, and CEUS-reported ETE between non-ETE and ETE groups (all *p* < 0.05), as shown in Table 2. Age, tumor size, BMUS-reported ETE, and CEUS-reported ETE were then introduced into the backward stepwise multivariate logistic regression analysis in order to build the clinical model (Table 3). The AUCs of the clinical models in the training and validation sets were 0.793 and 0.718, respectively (Table 4).

### 3.4. Clinical-Radiomics Model

**Figure 5:**
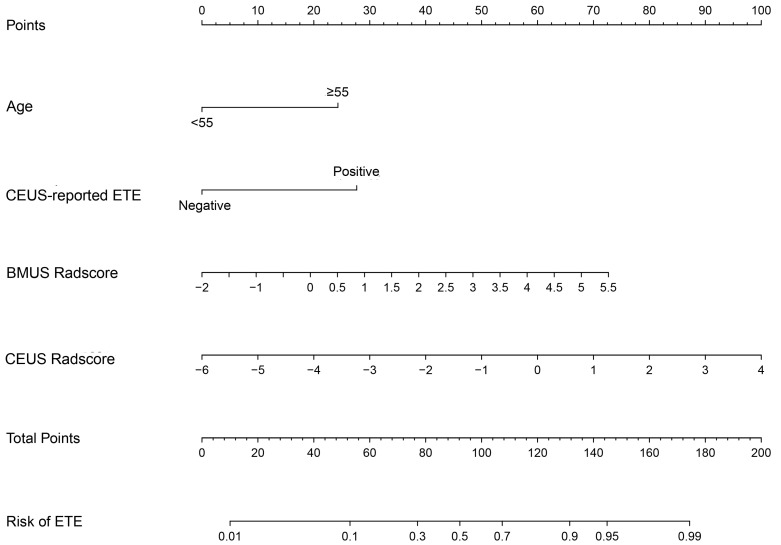
Developed clinical-radiomics nomogram. The clinical-radiomics nomogram was developed in the training group, with age, CEUS-reported ETE, BMUS Radscore, and CEUS Radscore. CEUS, contrast-enhanced ultrasound. ETE, extrathyroidal extension. BMUS, B-mode ultrasound.

**Figure 6:**
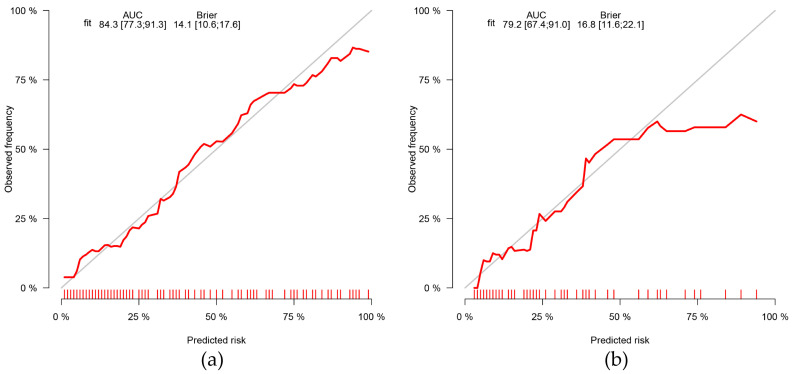
Calibration curves of the clinical-radiomics nomogram in the training (**a**) and validation (**b**) groups. The grey diagonal line in the curve represents a perfect prediction by an ideal model. The red line represents the performance of the nomogram, of which a closer fit to the diagonal line represents a better prediction. AUC, area under the curve.

**Figure 7:**
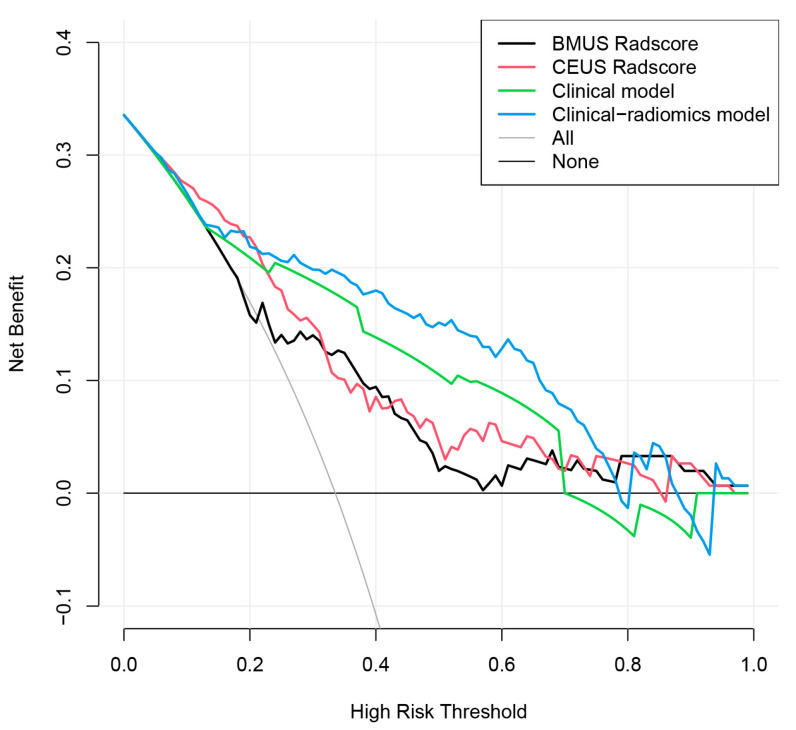
Decision curve analysis for the BMUS Radscore (black lines), CEUS Radscore (red lines), clinical model (green lines), and clinical-radiomics model (blue lines). The decision curve showed that if the threshold probability was 22–76%, then using the clinical-radiomics model to predict ETE has a greater advantage than using the BMUS Radscore, CEUS Radscore, and clinical model in terms of clinical applications. CEUS, contrast-enhanced ultrasound. BMUS, B-mode ultrasound.

The clinical risk factors, along with the two Radscores, were integrated to construct a clinical-radiomics model. According to the results of univariate analyses and multivariate stepwise logistic regression in the training set, age, CEUS-reported ETE, BMUS Radscore, and CEUS Radscore were finally included as indicators for constructing the combined model (Table 3). The clinical-radiomics model showed the highest predictive ability for the ETE of PTC in the training and validation sets with AUCs of 0.843 and 0.792, respectively (Table 4); therefore, it was identified as the best model. The AUC value of the clinical-radiomics model was significantly higher than that of the clinical model, both with respect to the training set (*p* = 0.004) and the validation set (*p* = 0.044). In addition, the clinical-radiomics model outperformed the BMUS Radscore (*p* < 0.001) and the CEUS Radscore (*p* = 0.009) in the training set. However, the performance of the clinical-radiomics model did not differ from that of the BMUS Radscore (*p* = 0.343) and CEUS Radscore (*p* = 0.419) in the validation set. The nomogram for visualizing the clinical-radiomics model is shown in Figure 5. The calibration curve analysis (Figure 6) and Hosmer–Lemeshow test for the clinical-radiomics model indicated proper agreement between the actual observed and predicted probabilities in the training set (*p* = 0.6466; Figure 6a) and the validation set (*p* = 0.2250; Figure 6b). Decision curve analysis was used to compare the clinical benefits of four prediction models, and we discovered that when the threshold probability was 22%-76%, the clinical-radiomics model was more beneficial than the clinical model and radiomics scores (Figure 7).

## 4. Discussion

In this study, BMUS and CEUS radiomics analyses were introduced for predicting ETE in PTC. Based on BMUS radiomics features, CEUS radiomics features, clinical risk factors, and a combination of them, we developed four prediction models for the preoperative prediction of ETE in PTC patients, which were subsequently validated and compared. We found that the clinical-radiomics model combining BMUS, CEUS radiomics features, and clinical risk factors had the best predictive efficacy (AUC of 0.843 in the training set and 0.792 in the validation set), and this was significantly better than the efficacy of the clinical model. The clinical-radiomics model provides an innovative solution for the pre-operative and personalized prediction of ETE.

Among eleven clinical factors, age and CEUS-reported ETE were found to be independent predictors of ETE in PTC. ETE usually occurs in PTC patients that are older than 55 years [37], and this also supports the finding in this study that the incidence of ETE was higher in older patients (age ≥ 55 years) than in younger patients (age < 55 years). In addition, imaging evaluation is essential for diagnosing ETE in patients with PTC. Ultrasound is the imaging examination of choice for evaluating thyroid nodules as it can clearly demonstrate the extent of contact between tumors and the adjacent thyroid capsule. In this study, radiologists predicted ETE based on BMUS and CEUS images. Both BMUS-reported ETE and CEUS-reported ETE were significantly associated with ETE in the univariate analysis, but interestingly, the BMUS-reported ETE did not enter the final clinical-radiomics model. We discovered that the more sufficient discriminatory power of CEUS-reported ETE weakened the weight of the BMUS-reported ETE. Previous studies have reported that CEUS has a higher diagnostic value for detecting ETE than BMUS [20,21], which is in line with our findings. We analyzed that the reasons for this may be that CEUS can show the blood supply difference between normal thyroid tissues and PTC, and it may also be more advantageous in showing the microvascularity of the thyroid capsule; thus, it is beneficial in showing the lesion’s outline and interruptions in capsule continuity. However, CEUS still has certain limitations. The interpretation of ultrasound images is highly subjective and dependent on the skill and experience of the radiologist. Therefore, there is a need for a more objective and accurate tool to help optimize the pre-operative prediction of ETE statuses in patients with PTC.

Radiomics is an emerging method of imaging analysis. The biological behavior and molecular biology associated with cancer are usually known via biopsies, which are invasive; in contrast, radiomics can be exploited as a rapid, cost-effective, and non-invasive imaging biomarker [38,39,40], and it may offer a promising alternative to biopsies for assessing tumors, such as those observed in breast cancer [41,42], thyroid cancer [43], and prostate cancer [44]. In addition, radiomics can overcome the subjectivity of traditional image diagnoses and provide quantitative imaging features [45,46,47], opening up a new tumor-imaging landscape [48]. Previous studies have shown that radiomics can be used to predict the ETE in PTC. Wang et al. established a US-based radiomics nomogram with radiomics features from BMUS images for the pre-operative prediction of ETE [36]. Both Chen et al. [49] and Yu et al. [50] reported that the CT-based radiomics nomogram significantly improved the pre-operative prediction of ETE. Xu et al. [51] found that iodine-map-based radiomics demonstrated better performance than that based on conventional CT images in predicting ETE. The iodine-map-based radiomics score complemented the clinical risk factors in predicting the ETE in PTC, and the clinical-radiomics nomogram outperformed the clinical model. Both He et al. [52] and Wei et al. [53] constructed radiomics models based on multiparametric MRI in pre-operatively predicting the ETE in patients with PTC. In the study of He et al., the combination of features from biparametric MRI images for analysis was limited by the small sample size (only 60 patients). Ran et al. collected T2WI, ADC, and contrast-enhanced T1-weighted (CE-T1WI) images from 132 patients with PTC. The established multiparametric MRI-based radiomics model contained 16 features, most of which were from T2WI images (seven features), and the most highly weighted feature was also from T2WI images, indicating that T2WI images provided more information. Additionally, the combination of multi-sequence MRI features provides more information than individually using a single sequence. This also provides insight and reference for our study: we can obtain more tumor information from more ultrasound images of different modalities.

Compared to the above radiomics investigations on similar topics, our study had some differences and improvements. We focused on the clinical value of radiomics features from ultrasound imaging in the prediction of ETE in PTC. Additionally, unlike previous ultrasound radiomics studies using only BMUS images [36], we constructed radiomics scores based on different ultrasound images (BMUS and CEUS images). Both BMUS Radscore and CEUS Radscore were associated with ETE in univariate analyses. After stepwise multivariate logistic regression, CEUS Radscore remained an independent predictor of ETE, and BMUS Radscore also entered the final model according to the backward stepwise selection with the AIC. CEUS Radscore had a higher odds ratio and higher AUC than BMUS Radscore, indicating that CEUS images may play more important roles in predicting ETE and provide additional information related to tumor perfusion compared to BMUS images. This is similar to the results of previously enhanced image-based radiomics research [51], which may correlate with increased tumor perfusion due to angiogenesis in aggressive tumors, and tumor heterogeneity in blood perfusions was highlighted by enhanced imaging, suggesting that tumors with strong vascular heterogeneity are more likely to develop ETE. We found the final selected features in BMUS Radcore and CEUS Radscore were all texture features. These textural features are associated with texture heterogeneity [54], which may correspond to the heterogeneity of tumor tissues and the vascular supply. PTC with ETE showed higher tumor heterogeneity.

Medical imaging-based clinical predictive models are a focus of research in precision medicine. They use multi-factor modeling to estimate the probability of a certain clinical event or a certain clinical outcome, including diagnoses and prognostic assessments [55]. Logistic regression or Cox regression are common methods for developing predictive models, but the regression formulas are not intuitive enough for clinical replication. Nomograms are graphic illustrations of statistically predictive models and are used for estimating individual patients’ specific risks [56,57,58]. Nomograms combining clinical risk factors and the radiomics score have been widely used to solve practical clinical problems. Our findings are consistent with many previous studies showing that the combination of clinical risk factors and radiomics improves a model’s performance, producing robust results [59,60,61,62]. In this study, a clinical-radiomics nomogram was constructed by integrating age, CEUS-reported ETE, BMUS Radscore, and CEUS Radscore. We found that the diagnostic performance of the nomogram model in predicting ETE was better than the radiomics score of a single ultrasound modality, indicating that the clinical-radiomics nomogram based on the dual-modal ultrasound contains more comprehensive and accurate tumor-related information. In addition, the clinical-radiomics nomogram had a significantly better AUC than the clinical model (*p*-value < 0.05 in both the training set and validation set), and its sensitivity and specificity were better than those of the clinical model, demonstrating that the addition of dual-modal ultrasound radiomics indicators improved the diagnostic performance.

This study has several limitations. First, this study is a single-center retrospective study with a relatively limited sample size; thus, further validation in prospective multicenter studies with large samples is needed in the future. Second, a single static image at the peak time of CEUS was selected for CEUS radiomics, and this study provides good confidence in the feasibility of CEUS images for radiomics analyses. However, the information contained in a single image may not be comprehensive enough, and further research on methods for obtaining more valuable information from CEUS videos is necessary in the future. Third, in terms of the CEUS enhancement degree, Chen et al. observed that invasive PTCs (presenting ETE or cervical lymph node metastasis) were more likely to exhibit hyperenhancement [20]; however, Liu et al. reported that there is no significant difference in the enhancement degree between the ETE and non-ETE groups [63]. Our study found that the enhancement degree was not associated with ETE, but our sample size was limited by a small number of hyper-enhancement samples, and the discrimination of the CEUS enhancement degree was influenced by personal experience. Currently, there is limited research assessing ETE by using the CEUS enhancement degree alone, which is controversial, and further studies with larger sample sizes are needed. Fourth, combining multiple imaging modalities (such as ultrasound, CT, and MRI), or combining multiple regions (such as intratumoral and peritumoral regions) for radiomics analyses may effectively expand the feature pool to yield more valuable diagnostic information and may be beneficial in improving the accuracy of diagnosing the disease.

## 5. Conclusions

We propose a clinical-radiomics nomogram integrating dual-modal ultrasound radiomics features and clinical risk factors. The nomogram supplements the traditional clinical strategy and can be exploited as a non-invasive pre-operative predictive tool for ETE in PTC patients.

## Figures and Tables

**Table 1:** Baseline patient characteristics in the training and validation sets.

Characteristics	Training Set (*n* = 152)	Validation Set (*n* = 64)	*p*-Value
Extrathyroidal extension			0.579
Negative	101 (66.4)	45 (70.3)	
Positive	51 (33.6)	19 (29.7)	
Age			0.622
<55 years	124 (81.6)	54 (84.4)	
≥55 years	28 (18.4)	10 (15.6)	
Gender			0.919
Female	113 (74.3)	48 (75.0)	
Male	39 (25.7)	16 (25.0)	
Primary site			0.537
Left lobe	66 (43.4)	31 (48.4)	
Right lobe	76 (50.0)	31 (48.4)	
Isthmus	10 (6.6)	2 (3.1)	
Tumor size			0.266
≤10 mm	100 (65.8)	37 (57.8)	
>10 mm	52 (34.2)	27 (42.2)	
Echogenicity			0.120
Iso/hyperechoic	6 (3.9)	5 (7.8)	
Hypoechoic	53 (34.9)	29 (45.3)	
Marked hypoechoic	93 (61.2)	30 (46.9)	
Aspect ratio > 1			0.381
Absent	95 (62.5)	44 (68.8)	
Present	57 (37.5)	20 (31.2)	
Margin			0.133
Smooth	6 (3.9)	7 (10.9)	
Ill-defined	20 (13.2)	9 (14.1)	
Irregular	126 (82.9)	48 (75.0)	
Microcalcification			0.222
Absent	43 (28.3)	13 (20.3)	
Present	109 (71.7)	51 (79.7)	
Enhancement degree			0.974
Hyper-enhancement	7 (4.6)	3 (4.7)	
Iso-enhancement	33 (21.7)	13 (20.3)	
Hypo-enhancement	112 (73.7)	48 (75)	
BMUS-reported ETE			0.886
Negative	80 (52.6)	33 (51.6)	
Positive	72 (47.4)	31 (48.4)	
CEUS-reported ETE			0.523
Negative	110 (72.4)	49 (76.6)	
Positive	42 (27.6)	15 (23.4)	
BMUS Radscore,			0.438
Median (interquartile range)	−1.01 (−1.29, −0.48)	−0.90 (−1.30, −0.35)	
CEUS Radscore,			0.317
Median (interquartile range)	−0.84 (−1.68, −0.11)	−0.64 (−1.29, −0.03)	

BMUS, B-mode ultrasound. ETE, extrathyroidal extension. CEUS, contrast-enhanced ultrasound.

**Table 2:** Associations between extrathyroidal extension and patient characteristics in the training and validation sets.

Characteristics	Training Set			Validation Set		
	ETE−	ETE+	*p*-Value	ETE−	ETE+	*p*-Value
Age			0.003			0.466
<55 years	89 (88.1)	35 (68.6)		39 (86.7)	15 (78.9)	
≥55 years	12 (11.9)	16 (31.4)		6 (13.3)	4 (21.1)	
Gender			0.252			0.530
Female	78 (77.2)	35 (68.6)		35 (77.8)	13 (68.4)	
Male	23 (22.8)	16 (31.4)		10 (22.2)	6 (31.6)	
Primary site			0.338			1.000
Left lobe	42 (41.6)	24 (47.1)		22 (48.9)	9 (47.4)	
Right lobe	54 (53.5)	22 (43.1)		21 (46.7)	10 (52.6)	
Isthmus	5 (5.0)	5 (9.8)		2 (4.4)	0 (0.0)	
Tumor size			0.002			0.006
>10 mm	75 (74.3)	25 (49)		31 (68.9)	6 (31.6)	
≤10 mm	26 (25.7)	26 (51)		14 (31.1)	13 (68.4)	
Echogenicity			0.527			0.271
Iso/hyperechoic	5 (5.0)	1 (2.0)		3 (6.7)	2 (10.5)	
Hypoechoic	37 (36.6)	16 (31.4)		18 (40)	11 (57.9)	
Marked hypoechoic	59 (58.4)	34 (66.7)		24 (53.3)	6 (31.6)	
Aspect ratio > 1			0.690			0.531
Absent	62 (61.4)	33 (64.7)		32 (71.1)	12 (63.2)	
Present	39 (38.6)	18 (35.3)		13 (28.9)	7 (36.8)	
Margin			0.467			0.108
Smooth	4 (4.0)	2 (3.9)		3 (6.7)	4 (21.1)	
Ill-defined	11 (10.9)	9 (17.6)		5 (11.1)	4 (21.1)	
Irregular	86 (85.1)	40 (78.4)		37 (82.2)	11 (57.9)	
Microcalcification			0.355			0.739
Absent	31 (30.7)	12 (23.5)		10 (22.2)	3 (15.8)	
Present	70 (69.3)	39 (76.5)		35 (77.8)	16 (84.2)	
Enhancement degree			0.230			0.298
Hyper-enhancement	5 (5.0)	2 (3.9)		2 (4.4)	1 (5.3)	
Iso-enhancement	26 (25.7)	7 (13.7)		7 (15.6)	6 (31.6)	
Hypo-enhancement	70 (69.3)	42 (82.4)		36 (80)	12 (63.2)	
BMUS-reported ETE			0.019			0.038
Negative	60 (59.4)	20 (39.2)		27 (60.0)	6 (31.6)	
Positive	41 (40.6)	31 (60.8)		18 (40.0)	13 (68.4)	
CEUS-reported ETE			<0.001			0.117
Negative	88 (87.1)	22 (43.1)		37 (82.2)	12 (63.2)	
Positive	13 (12.9)	29 (56.9)		8 (17.8)	7 (36.8)	
BMUS Radscore			<0.001			0.004
Median (interquartile range)	−1.11 (−1.31, −0.85)	−0.56 (−1.13, 0.05)		−0.97 (−1.31, −0.61)	−0.36 (−0.93, 0.45)	
CEUS Radscore			<0.001			0.003
Median (interquartile range)	−1.28 (−2.00, −0.48)	−0.23 (−0.83, 0.52)		−0.97 (−1.79, −0.28)	−0.09 (−0.60, 0.21)	

BMUS, B-mode ultrasound. ETE, extrathyroidal extension. CEUS, contrast-enhanced ultrasound.

**Table 3:** Four prediction models based on stepwise multivariate analyses for the prediction of ETE.

Characteristics	Odds Ratio (95%CI)	*p*-Value
BMUS radiomics model		
original_ngtdm_Busyness	2.58 (1.60, 4.15)	<0.001
CEUS radiomics model		
wavelet.LHL_glszm_SmallAreaEmphasis	1.87 (1.14, 3.08)	0.013
wavelet.HLL_gldm_DependenceVariance	0.50 (0.31, 0.79)	0.003
wavelet.HLL_ngtdm_Complexity	1.70 (1.11, 2.61)	0.015
Clinical model		
Age (≥55 years vs. <55 years)	4.00 (1.52, 10.50)	0.005
Tumor size (>10 mm vs. ≤10 mm)	2.08 (0.90, 4.81)	0.087
CEUS-reported ETE (positive vs. negative)	7.42 (3.14, 17.56)	<0.001
Clinical-radiomics model		
Age (≥55 years vs. <55 years)	3.89 (1.45, 10.49)	0.007
CEUS-reported ETE (positive vs. negative)	4.70 (1.84, 11.99)	0.001
BMUS Radscore	1.72 (0.98, 3.01)	0.058
CEUS Radscore	1.75 (1.09, 2.80)	0.020

CI, confidence interval. BMUS, B-mode ultrasound. CEUS, contrast-enhanced ultrasound. ETE, extrathyroidal extension.

**Table 4:** Performance of four models in the training and validation sets.

Group	Model	AUC (95% CI)	*p* Value (vs. Combined Model)	Sensitivity	Specificity	Cutoff Value
Training set	BMUS radiomics	0.704 (0.610–0.799)	<0.001	0.627	0.782	0.305
	CEUS radiomics	0.768 (0.694–0.843)	0.009	0.922	0.495	0.213
	Clinical	0.793 (0.715–0.871)	0.004	0.745	0.782	0.307
	Clinical-radiomics	0.843 (0.773–0.913)	-	0.765	0.832	0.356
Validation set	BMUS radiomics	0.731 (0.595–0.867)	0.343	0.632	0.689	0.305
	CEUS radiomics	0.739 (0.617–0.861)	0.419	0.947	0.333	0.213
	Clinical	0.718 (0.588–0.847)	0.044	0.526	0.711	0.307
	Clinical-radiomics	0.792 (0.674–0.910)	-	0.789	0.778	0.356

CI, confidence interval. AUC, area under the curve. BMUS, B-mode ultrasound. CEUS, contrast-enhanced ultrasound.

## Data Availability

The original contributions presented in the study are included in the article, and further inquiries can be directed to the corresponding author.
